# Impact of COVID-19 on the vascular interventionist

**DOI:** 10.1177/1708538120930141

**Published:** 2020-06-02

**Authors:** Albeir Y Mousa, Mike Broce

**Affiliations:** 1 Department of Surgery, Robert C. Byrd Health Sciences Center, West Virginia University, Charleston, WV, USA; 2 Vascular Center of Excellence, Charleston Area Medical Center, Charleston, WV, USA; 3 Center for Health Services and Outcomes Research, Charleston Area Medical Center, Health Education and Research Institute, Charleston, WV, USA

The COVID-19 pandemic considerably distorted all of our lives personally and professionally. Personally, we strive to maintain safety measures for ourselves and families. Still, we have to practice within strict guidelines dictated by national and local authorities for the ultimate protection of health care professionals and patients, which required elective surgeries to be postponed indefinitely, patients with initial and follow-up clinic visits placed on hold, and hospital resources redistributed.

COVID-19 impacted:

*Operative time*: The number of surgeries decreased to only urgent or emergent cases. This may create a double-edge sword, with increased demand and cost generated to maintain a COVID-free environment along with a remarkable decrease in cash flow to the hospital.
*SVS VQI*: The disruption of routine patient flow will cause a delay in data entry for new and follow-up visits, which will result in visits outside of the prescribed time period (9–21 months).
*Clinical trials*: Many centers with enough volume participate in clinical trials. The sharp decrease in elective procedures along with restricted clinic time will lower the number of patients available for trials. Consequently, such clinical trials are now looking to increase days of enrollment and to rely on electronic (E)-clinic visits to obtain some sort of follow-up.
*Vascular training*: Vascular surgery board is accepting 44 weeks of clinical time and 10% decrease in total cases for current academic year. Medicare has modified their policy and waived supervision for urgent procedures in critical ill cases.
*Vascular lab*: The number of cases parallels the decrease in clinic visits. Many other issues arise related to safety of the imaging and vision laboratory.
*Vascular conferences*: Without exception all regional and national meetings have been canceled, and societies postponed their abstract due dates. For the first time in history, the Vascular Annual Meeting in Canada was canceled.
*Current practice*: Vascular interventionist are encouraged to learn about ventilators and asked to help in intensive care units. Personal protective equipment (PPEs) for all health care members is a must, but adds greatly to routine practice, while most institutions strive to carry sufficient inventory.
*Legislation*: Many states have waived any required license to encourage physicians to move to areas with desperate needs.
*Payment*: Medicare has issued many regulatory payments to encourage remote E-visits.
*Call schedule*: This has been limited to cover only emergencies, and it is imperative to remotely connect to patients for immediate, follow-up and future treatment normally through E-visits.

In conclusion:

The unprecedented COVID-19 pandemic impacts our vascular specialty. Still, it is positively alternating the routine of our daily practices.

First and far most, we should focus on the safety of our patients by screening measurements to rule out fever, cough, and dyspnea and thereby creating a safety triage area with competent screening measures to stratify patients to appropriate care.

Reinforcing the CDC safety guidelines such as wearing appropriate PPE, gown, gloves, eye protection, and N95 respirator mask to prevent any contact with droplets/aerosols as the virus may stay active on surface for 72 h. Along with social distance as 50% of confirmed COVID-19 patients reported exposure to infected individuals.

Evolving telemedicine services comes to play a huge part when it comes to E-visit our patients at their homes/residences. Multiple clinical trials are recruiting and will update their outcomes completion. Virtual vascular conferences across the board should continue with our vascular trainees. Our chief vascular residents and fellows should be acquainted with the SVS updated COVID-19 guidelines for their required case-log and clinical time.

Vascular specialists should reach out to our patients to determine urgent and emergent cases. I would suspect that soon enough we will increase our practice scoop to operate on urgent cases with whom we have a large portion in our practices.

There are ongoing sweeping regulations from CMS to empower health providers and institutions during the current COVID-19 surge. These legislations include licensures, payments, and reimbursements.

